# Service disengagement in first episode psychosis: rates and predictors form 2-year longitudinal research in a real-world care setting

**DOI:** 10.1192/j.eurpsy.2025.330

**Published:** 2025-08-26

**Authors:** L. Pelizza, E. Leuci, E. Quattrone, M. Menchetti, F. Catalano

**Affiliations:** 1 Università di Bologna, Bologna; 2 AUSL di Parma, Parma; 3 University of Bologna Italy, Bologna, Italy

## Abstract

**Introduction:**

Service disengagement is a major problem for “Early Intervention in Psychosis” (EIP). Understanding predictors of engagement is also crucial to increase effectiveness of mental health treatments, especially in young people with First Episode Psychosis (FEP). No Italian investigation on this topic has been reported in the literature to date. The goal of this research was to assess service disengagement rate and predictors in an Italian sample of FEP subjects treated within an EIP program across a 2-year follow-up period.

**Objectives:**

The goal of this research was to assess service disengagement rate and predictors in an Italian sample of FEP subjects treated within an EIP program across a 2-year follow-up period.

**Methods:**

All patients were young FEP help-seekers, aged 12–35 years, recruited within the “Parma Early Psychosis” (Pr-EP) program. At baseline, they completed the Positive And Negative Syndrome Scale (PANSS) and the Global Assessment of Functioning (GAF) scale. Univariate and multivariate Cox regression analyses were carried out.

**Results:**

489 FEP subjects were enrolled in this study. Across the follow-up, a 26 % prevalence rate of service disengagement was found. Particularly strong predictors of disengagement were living with parents, poor treatment adherence at entry and a low baseline PANSS “Disorganization” factor score.

**Conclusions:**

More than a quarter of our FEP individuals disengaged the Pr-EP program during the first 2 years of intervention. A possible solution to reduce disengagement and to facilitate re-engagement of these young patients might be to offer the option of low-intensity monitoring and support, also via remote technology and telemental health care.

**Disclosure of Interest:**

None Declared

